# Use of modified lateral upper arm free flap for reconstruction of soft tissue defect after resection of oral cancer

**DOI:** 10.1186/s13005-016-0105-1

**Published:** 2016-01-29

**Authors:** Xu-Dong Yang, Su-Feng Zhao, Qian Zhang, Yu-Xin Wang, Wei Li, Xiao-Wei Hong, Qin-Gang Hu

**Affiliations:** Department of Oral and Maxillofacial Surgery, Nanjing Stomatological Hospital, Medical School of Nanjing University, No. 30 Zhong Yang Rd, Nanjing, 210008 People’s Republic of China

**Keywords:** Extended lateral upper arm free flap, Oral cancer, Soft tissue defect, Repair, Anastomosis

## Abstract

**Background:**

To evaluate the suitability of a modified lateral upper arm free flap (LAFF) for reconstruction of soft tissue defects after resection of oral cancer.

**Methods:**

Eighteen cases of soft tissue defect repair performed between January 2011 and December 2013 using a modified LAFF after resection of oral cancer were reviewed. The design and harvest of the LAFF, the reconstruction procedure, and postoperative morbidity were reviewed and evaluated over a follow-up period of at least 12 months.

**Results:**

The overall flap survival was 94.4 % (17/18 patients). A broad scar at the donor site was the most common morbidity, but patients did not report dissatisfaction with the scar because they could easily cover it. All wounds at the donor site achieved primary recovery. One case of flap loss was repaired with a radial forearm free flap. One case complicated by diabetes mellitus involved infection of the flap with one-third of the flap becoming necrotic. This flap survived after removal of the necrotic tissue. In one other case, fat liquefactive necrosis (1.5 × 1.0 cm) occurred in the flap on the tip of the tongue, and this flap survived after debridement. Overall, the shape and function of the reconstructed tissues were well restored, and there was no severe morbidity at the donor site in any case.

**Conclusion:**

The modified LAFF was safe and reliable for the reconstruction of soft tissue defects after resection of oral cancer.

## Background

Oral cancer is the 11^th^ most common cancer type worldwide, with a still increasing incidence, and it is typically associated with squamous cell carcinoma and a high risk of metastases to lymph nodes in the neck [1, 2]. Surgical resection is an appropriate treatment strategy for oral cancer [3, 4]. However, tissue defects that are created by resection of oral cancer can severely affect speech and swallowing as well as disturb the aesthetic appearance of the oral cavity. A variety of free tissue flaps have been researched and then widely used in the reconstruction of tumor-related defects in the oral cavity [5], with the most common flaps included the anterolateral tight flap and the pectoralis major and latissimus dorsi muocutaneous flap. Unfortunately, these flaps are bulky and not always suitable for restoring the function of the delicate oral anatomy [5]. In 1982, Song et al. [6] introduced the lateral upper arm free flap (LAFF), which has since been used in a variety of anatomical reconstruction procedures for areas including the head and neck because it is a thin, soft, and sensory tissue flap that offers a suitable amount of tissue and color for reconstruction as well as low morbidity at the donor site [5–8]. However, the utility of the LAFF in the repair of oral defects following resection of oral cancer has not been established in the literature. Therefore, in the present study, we report the outcomes of 18 cases in which the modified LAFF was applied to reconstruct a tissue defect after radical resection of oral cancer.

## Methods

### Clinical data

From January 2011 to December 2013, 18 patients with cancer of the oral cavity first underwent radical resection of the cancer and then intraoral reconstruction using a modified LAFF in the Department of Oral and Maxillofacial Surgery, Affiliated Stomatological Hospital, Nanjing University Medical School. The preoperative assessment included clinical examination of the donor site by an experienced surgeon. The study was approved by Ethical institutional review board of Stomatological Hospital of Nanjing University School of Medicine (NO. LC 2010-17/1).

### LAFF anatomy

The conventional LAFF is situated on the lower lateral aspect of upper arm immediate above the medial condyle of humerus and is a typical intermuscular septum flap. The posterior radial collateral artery (PRCA) as well as the deep brachial artery and its branches are the main feeding vessels of the LAFF, according to the intermuscular septum of the lateral arm. The deep brachial artery originates from the proximal lateral segment of the branchial artery and runs close to the radial nerve in a spiral groove and then bifurcates into the PRCA and anterior radial collateral artery. The PRCA runs in the septum between the triceps, brachialis, and brachioradialis, and a number of branches extending from the PRCA feed the lateral head of the triceps. Upon harvesting the LAFF, the terminal of the PRCA was anastomosed with the terminal of arteria radialis recurrens around the humeral lateral epicondyle, which composed the vascular network around the humeral lateral epicondyle. The range of blood supply could extend 8 cm distally. These features are the anatomic basis for the harvest and modification of the LAFF.

### LAFF harvesting

**Fig. 1:**
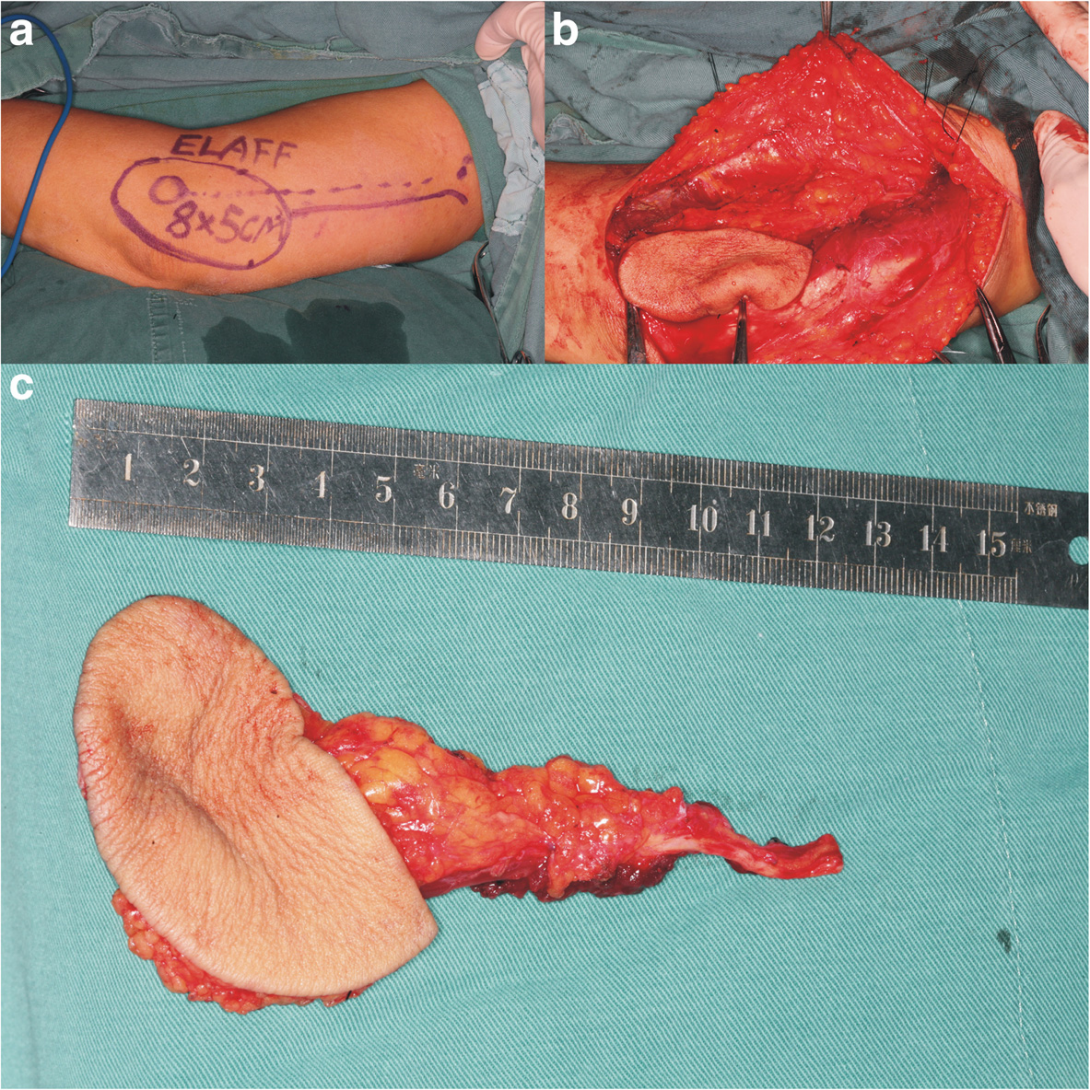
**a** Representative image showing the LAFF design with the lateral intermuscular septum as the central axis and the lateral condyle at the inferior trisection point of flap. The flap had the shape of an ellipse with a width of no more than 6 cm. **b** Representative image during harvesting of the LAFF showing both the anterograde and retrograde anatomy. **c** Representative image of a free cutaneous flap with a 10.2-cm vascular pedicle

A color ultrasonic Doppler blood flow survey meter was used to examine the left upper arm to exclude variations in the septocutaneous perforators preoperatively while all patients were under general anesthesia. A line was marked between the deltoid insertion and the lateral condyle, and 1 cm behind which indicated the lateral intermuscular septum and PRCA. The flap had the shape of an ellipse and a width of no more than 6 cm (Fig. 1a). The free flaps were harvested with the combination of anterograde and retrograde tracing without blood evacuation (Fig. 1b). First, the vascular pedicle was isolated. The front of the lateral triceps head was isolated at the junctional part of the deltoid muscle and triceps muscle after the skin and subcutaneous tissue were dissected at the base of the line. Next, the muscle was pulled back and the vascular pedicle was carefully isolated proximally before the radial collateral artery was isolated to the extendible portion of the deep brachial artery. Because the radial nerve might be touched during the procedure, the vascular was isolated precisely by lifting with moderate tension of a rubber band, which could avoid injury to the radial nerve. The anterior radial collateral artery was ligatured, and then the flap was isolated until the posterior radial collateral artery and the parallel vein of the vascular pedicle. Secondly, the posterior incision was made, and the skin, subcutaneous tissue, and triceps fascia were cut. Then the tracing proceeded along the muscle surface to the lateral intermuscular septum. Then, the septocutaneous perforators in the intermuscular septum were identified, and close attention was paid to protecting these from injury. The main vascular pedicle of the flap PRCA and its parallel vein were preserved by continuing to isolate the intermuscular septum to its bottom. The front and distal ends of the flap were cut as deep as the brachioradialis and brachial muscle fascia after the vascular pedicle was isolated to its deep surface, and then the front of the lateral intermuscular septum was identified after isolation along the muscular belly. The flap was designed with the lateral intermuscular septum as the central vascular axis and the lateral condyle located at the inferior third point. The flap then extended below the lateral condyle by approximately 3–4 cm and extended to the upper part of the forearm. Finally, the flap was well prepared after isolation with the vascular retrograde anatomy directed upward to the upper part. The vascular pedicles for reconstruction were at least 6 cm in length. The muscle tissue of the lateral triceps head could be included in the flap when a complex lateral arm flap was needed (Fig. 1c).

### Defect reconstruction and postoperative evaluation

The follow-up period was at least 12 months in all patients.

## Results

This study included 16 male patients (88.9 %) and 2 female patients (11.1 %) with a mean age of 54.8 years (range, 30–72 years). Among the 18 cases analyzed in this study, there were 17 cases of squamous cell carcinoma and 1 case of adenoid cystic carcinoma, and the cancers were located on the tongue (10 cases), pars buccalis (6 cases), and palate (2 cases). The mean LAFF size was 6.7 × 5.1 cm, and the mean length of the vascular pedicle was 9.5 cm. The thickness of the skin and subcutaneous tissue ranged from 7–15 mm (mean, 10.7 mm), and the perforators were present with no variation between flaps. Seven patients received adjuvant radiotherapy early in the postoperative period after the surgical wound had healed completely. The purpose of this delay was to ensure that poor functional outcomes could be correlated with postoperative adjuvant radiotherapy.

**Fig. 2:**
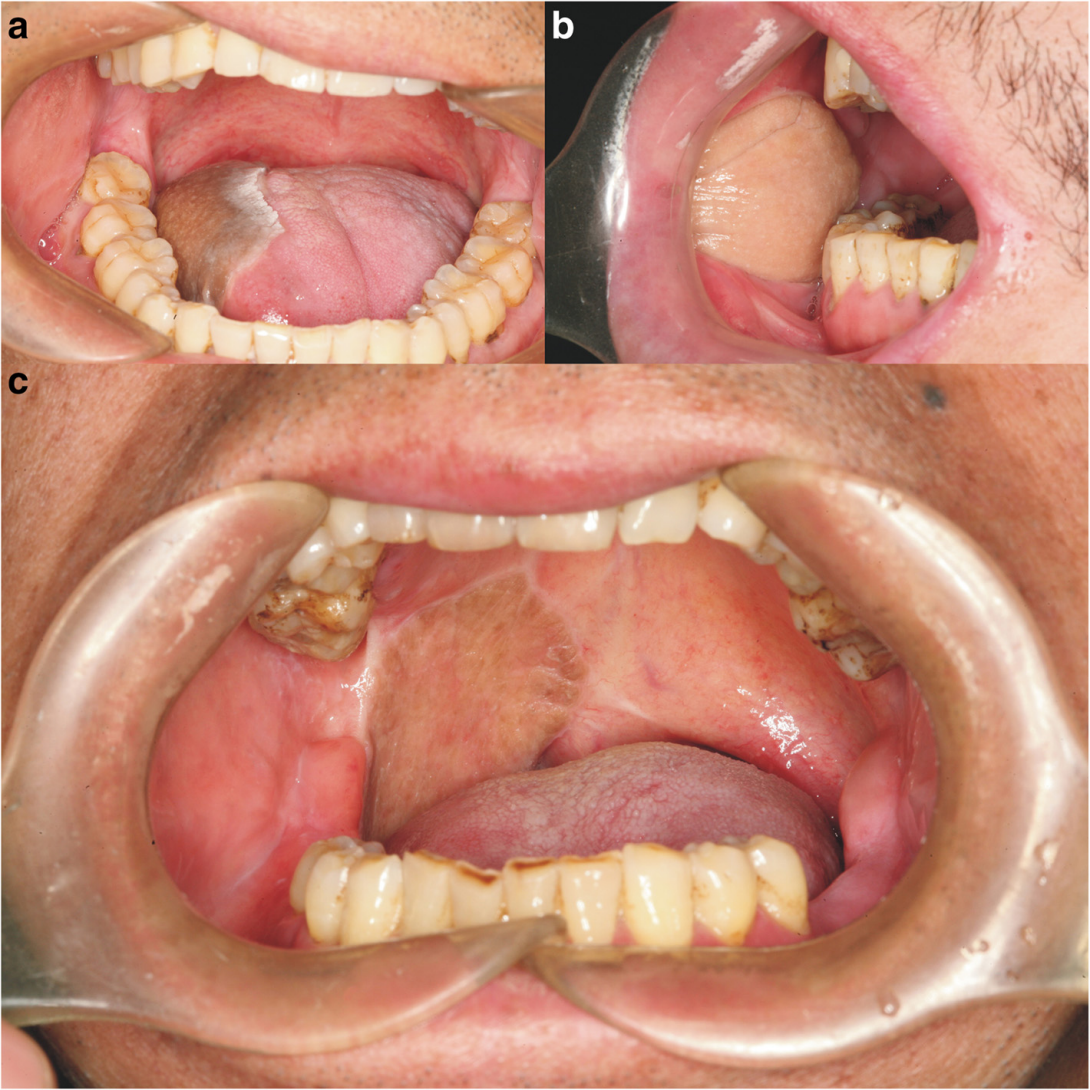
**a** Image of a LAFF transferred to the tongue of a patient at 6 months postoperatively, showing the soft texture and appropriate shape of the flap in a patient who recovered near-normal tongue movement. **b** Image of the transferred flap at 6 months postoperatively, showing the soft texture and clear dermatoglyph. **c** Image of the oral cavity of a patient at 6 months after transfer of a LAFF that healed well and provided good recovery of speech and swallowing functions

The mean surgery time was 7.4 h, and the mean blood loss was 550 ml. The overall survival rate of the transferred flaps was 94.4 % (17/18). One patient experienced total loss of the flap and underwent a reparative procedure using a radial forearm free flap. In one other case complicated by diabetes mellitus, the flap became infected with *Staphylococcus aureus* β and one-third of the flap became necrotic. However, the flap survived after pruning of the necrotic front end. In one case, fat liquefactive necrosis (1.5 × 1.0 cm) was observed in the flap on the tip of the tongue, and the flap survived after debridement. Figure 2a, b, c shows representative images of reconstructed defects 6 months after resection of tongue cancer, buccal carcinoma, and carcinoma of the palate.

The artery was end-to-end anastomosed to the external maxillary artery in 7 cases and with the superior thyroid artery in 11 cases. The parallel vein was end-to-side anastomosed to the internal jugular vein in all cases. An additional second vein was added in 7 cases, including the common facial vein in 5 cases and the external jugular vein in 2 cases. The ratio of vein-to-vein to artery-to-artery anastomosis was 1.39 to 1. In addition, in three patients with tongue cancer, the flap carried the posterior brachial cutaneous nerve, which was anastomosed to the stump of the lingual nerve.

**Fig. 3:**
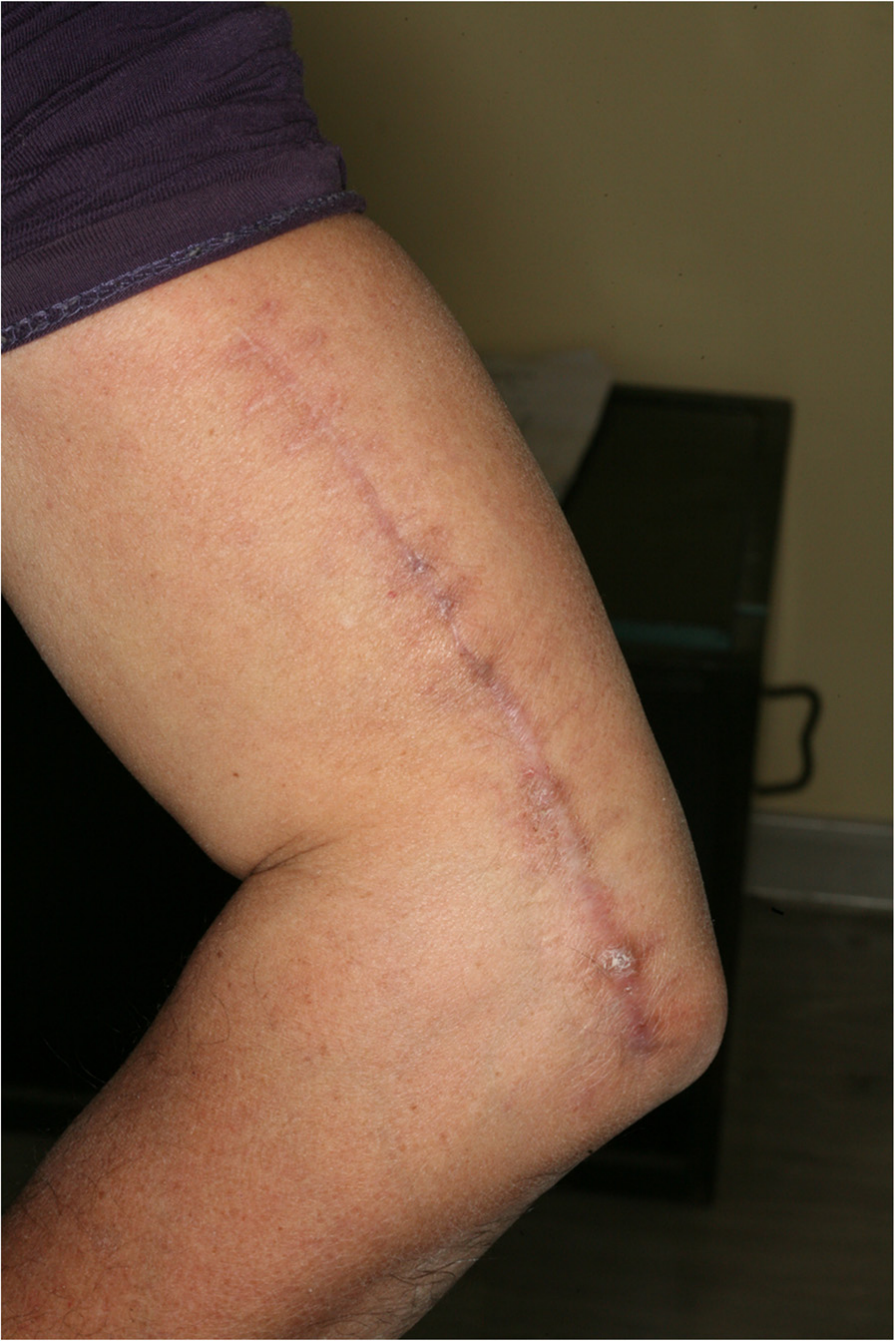
Representative image of the donor site at 6 months postoperatively, showing the absence of severe morbidity in a patient who experienced only mild hypoesthesia

As shown in the representative image in Fig. 3, the wound at the donor site in all cases achieved primary healing with no arm dysfunction, no necrosis of the skin and muscle tissue, and no severe scarring. Five cases experienced sensory disturbance at the proximal end of the lateral forearm (hypoesthesia in 2, paresthesia in 1, and hyperesthesia in 2), and two cases reported mild pain at the lateral condyle.

## Discussion

The ideal flap for soft tissue reconstruction in the oral cavity following oral cancer resection must not only offer aesthetically acceptable repaired tissue but also be able to restore speech and swallowing functions. Specifically, the donor site should provide a sufficient amount of tissue to permit easy harvest of the flap. The pedicle must be long enough to reach recipient vessels, and the vessels should long enough and large sufficiently for easy anastomosis. Closure of the donor site should be easy, with minimal morbidity after harvesting of the flap. The availability of a sensory cutaneous nerve is beneficial when reinnervation is required for functional recovery, and last but not least, the donor site should be in a location that would allow for simultaneous harvesting of tissue to repair head and neck defects [5, 9]. Based on these extensive requirements of the perfect donor site and flap for soft tissue reconstruction in the oral cavity, reconstructive surgeons have had difficulty choosing the most appropriate donor site and flap [9] and evaluating flaps against the criteria for an ideal flap.

A variety of tissue flaps have been applied to soft tissue repair after resection of oral cancer, including a forearm flap, the anterolateral tight flap, and the latissimus pectoris flap [10]. Each of these flaps meets some criteria for an ideal flap, but typically at the expense of other criteria. Thus, none of them can meet all of the criteria. Harvesting of the forearm flap requires severing the radial artery, which is the main feeding artery of the forearm. Moreover, with the use of this flap, the sense and motor function of hand as well as the appearance of the operative wound have not been satisfactory. The anterolateral tight flap and latissimus pectoris flap include too much fat tissue, making them rather bulky for most medium-size defects.

The use of the LAFF was introduced first by Song et al. [6] and then further described by Katsoros et al. [11]. The LAFF has been applied in the reconstruction of not only defects in the oral cavity [5, 12–14], but also defects in the pharyngeal cavity, lateral defects of temporal bone [15], lateral defects at the skull base [16], and defects resulting from parotidectomy [17], and these studies reported advantages of low morbidity at the donor site, moderate thickness of the flap, and excellent plasticity. However, the application of the conventional LAFF was limited due to the short pedicle and small vessel size. These disadvantages were overcome by application of the extended lateral upper arm free flap (ELAFF) introduced by Kuek and Chuan [18], which extended the inferior margin of the conventional LAFF to the upper part of the forearm by designing the inferior margin of the modified LAFF to be located at the attachment point of the deltoid. Compared with the conventional LAFF, the ELAFF offers enhanced texture, a longer vascular pedicle, and similar flexibility in the forearm. A previous study demonstrated that the ELAFF can meet most of the criteria for an ideal flap for soft tissue reconstruction in defects of the head and neck [9].

In the cases described in this study, we modified the conventional procedure for harvesting the ELAFF that was reported previously [19, 20]. In the modified design of the LAFF used in our study, the flap was extended distally below the lateral condyle, making the modified LAFF 3–4 cm longer than the conventional LAFF. A position too distal might leave a conspicuous scar, but a previous systematic review demonstrated that scar formation did not cause much patient dissatisfaction [7]. In our study, the linear scar at the donor site could be covered with appropriate dressing. The vascular pedicle was usually 7–9 cm when it was cut off at the terminus of the deltoid. Therefore, there was no need to extend the vascular pedicle, because it was already long enough, which served to simplify the operative procedure. The width of the modified LAFF was no larger than 6 cm, which permitted direct suturing and facilitated primary recovery of the donor site in all cases. Moreover, additional incisions could be applied if the donor site cannot be closed directly due to high tension. The proximal vascular pedicle was isolated first because it was not under excessive tensile stress, which could reduce injury to the vasculature in the flap. A previous study [21] demonstrated that sensory disturbance was the most common morbidity (61.4 %) at the LAFF donor site, and we speculated that this may be related to nerve injury. Therefore, harvesting of the modified LAFF was performed in the absence of a tourniquet, which avoided squeeze-induced injury to the radial nerve. To further avoid injury to the radial nerve, the vascular pedicle was isolated precisely under the moderate tensile force of a rubber band. One study showed that numbness and hypoesthesia can occur even when the posterior arm or forearm cutaneous nerve was preserved, but that these symptoms would resolve gradually [22]. The patients in our study who reported pain at the lateral epicondyle and hypoesthesia at the lateral aspect of the proximal forearm also reported that these symptoms gradually resolved. Finally, the use of LAFFs containing a vascularized posterior brachial cutaneous nerve as a sensory flap to reconstruct hemi-tongue defects and an anterior brachial cutaneous nerve of the LAFF anastomosed with a facial nerve for reconstruction of soft tissue defect after the resection of parotid carcinoma was reported previously. In three cases, the posterior brachial cutaneous nerve was anastomosed to the stump of the lingual nerve, but the effectiveness of this approach remains to be determined. The majority of soft tissue defects in the oral cavity need to be repaired with a composite flap including a portion of the muscle of the lateral head of the triceps, because the defects usually involve defects in both the muscle tissue and mucosa.

In the repair of tongue defects, the thin distal part of the modified LAFF (forearm part) was used to repair defects on the front-end of the tongue, while triceps muscle flaps were used to repair muscle defects at the floor of the mouth and the base of the tongue. Also, the proximal part of the modified LAFF was used to cover defects at the floor of the mouth and the base of the tongue. Liquefactive necrosis at the tip of tongue occurred in one female patient, which might be related to the bulk of the flap and the high tensile stress at the tip of the tongue. In another case complicated by diabetes mellitus, the flap became infected but recovered completely after resection of the forepart of the flap. In this case, the symptoms of itching, sensory disturbance, and cold intolerance at the upper arm were considered to be related to hyperglycemia.

Although the modified LAFF offers plenty of advantages, the available volume of tissue and the vessels available for anastomosis were reported to be major limitations to its widespread application [20]. The vessels of the LAFF are certainly smaller than those of a forearm flap, and the PRCA, which is a terminal branch of the deep brachial artery, is the main feeding vessel of the LAFF. Two main venous systems are present in this flap area: the deep venous system that travels along the PRCA and the subcutaneous network that is not involved in draining the flap. The diameters of the artery and vein of the upper arm are similar at 1.1 mm (range, 0.8–1.4 mm) and 1.2 mm (range, 0.9–1.5 mm) on average, respectively [23]. Thus, the artery and vein must be labeled during flap harvest. The external maxillary artery was isolated to the submandibular gland, to harvest the artery which diameter is similar with the PRCA. Considering that the diameter of the superior thyroid artery becomes gradually smaller, we recommend the superior thyroid artery as the first choice.

Moreover, sufficient blood supply at the donor site was achieved after PRCA anastomosis. The diameter of the vein that ran with the PRCA was much smaller than that of the superior thyroid vein or external maxillary vein. Thus, end-to-side anastomosis with the internal jugular vein was applied and a second vein anastomosis was added in some cases, which offered a more reliable and effective approach. The Allen test was not used to detect the PRCA because it was not main feeding vessel of the flap, and only a color ultrasonic Doppler blood flow survey meter was used to detect the PCRA and its branches and exclude variation.

Specific limitations of the present study include the lack of explicit inclusion and exclusion criteria for patients. Generally, the volume of the LAFF varies by gender, age, and weight. Flaps harvested from females are superior to those from males in terms of color, but flaps from female patients have more cutaneous fat [5]. Although flaps from female patients may better match the donor site, they may be less suitable for transfer in older patients, who might have thinner subcutaneous tissue and less hair compared to young patients [5]. Based on such expected variations in flap characteristics, we cannot draw decisive conclusions regarding the use of the modified LAFF for reconstructing soft tissue defects in the oral cavity, and further studies need to be performed.

## Conclusion

In summary, the modified ELAFF was found to be safe and suitable for the reconstruction of soft tissue defects after resection of oral cancer, especially for medium size defects, and therefore, we propose that the modified ELAFF provides a well-vascularized and reliable donor flap for this purpose.

## References

[1] Kelner N, Vartanian JG, Pinto CAL, Coutinho-Camillo CM, Kowalski LP (2014). Does elective neck dissection in T1/T2 carcinoma of the oral tongue and floor of the mouth influence recurrence and survival rates?. Brit J Oral Maxillofac Surg.

[2] Wang YY, Tail YH, Wang WC, Chen CY, Kao YH, Chen YK (2014). Malignant transformation in 5071 southern Taiwanese patients with potentially malignant oral mucosal disorders. BMC Oral Health.

[3] Tesseroli MA, Calabrese L, Carvalho AL, Kowalski LP, Chiesa F (2006). Discontinuous vs. in-continuity neck dissection in carcinoma of the oral cavity. Experience of two oncologic hospitals. Acta Otorhinolaryngol Ital.

[4] Liu J, Wu H, Zhu Z, Wu X, Tan H, Wang K (2010). Free anterolateral thigh myocutaneous flap for reconstruction of soft tissue defects following en block resection of tongue cancer. Zhongguo Xiu Fu Chong Jian Wai Ke Za Zhi.

[5] Song XM, Yuan Y, Tao ZJ, Wu HM, Yuan H, Wu YN (2007). Application of lateral arm free flap in oral and maxillofacial reconstruction following tumor surgery. Med Princ Pract.

[6] Song R, Song Y, Yu Y, Song Y (1982). The upper arm free flap. Clin Plast Surg.

[7] Thankappan K, Kuriakose MA, Chatni SS, Sharan R, Trivedi NP, Vijayaraghavan S (2011). Lateral arm free flap for oral tongue reconstruction: an analysis of surgical details, morbidity, and functional and aesthetic outcome. Ann Plast Surg.

[8] Agostini T, Lazzeri D, Spinelli G (2013). Anterolateral thigh flap: systematic literature review of specific donor-site complications and their management. J Craniomaxillofac Surg.

[9] Ross DA, Thomson JG, Restifo R, Tarro JM, Sasaki CT (1996). The extended lateral arm free flap for head and neck reconstruction: the Yale experience. Laryngoscope.

[10] Schusterman MA, Miller MJ, Reece GP, Kroll SS, Marchi M, Goepfert H (1994). A single center’s experience with 308 free flaps for repair of head and neck cancer defects. Plast Reconstr Surg.

[11] Katsaros J, Schusterman M, Beppu M, Banis JC, Acland RD (1984). The lateral upper arm flap: anatomy and clinical applications. Ann Plast Surg.

[12] Haas F, Seibert FJ, Koch H, Hubmer M, Moshammer HE, Pierer G (2003). Reconstruction of combined defects of the Achilles tendon and the overlying soft tissue with a fascia lata graft and a free fasciocutaneous lateral arm flap. Ann Plast Surg.

[13] Gellrich NC, Kwon TG, Lauer G, Fakler O, Gutwald R, Otten JE (2000). The lateral upper arm free flap for intraoral reconstruction. Int J Oral Maxillofac Surg.

[14] Vico PG, Coessens BC (1997). The distally based lateral arm flap for intraoral soft tissue reconstruction. Head Neck.

[15] Moncrieff MD, Hamilton SA, Lamberty GH, Malata CM, Hardy DG, Macfarlane R (2007). Reconstructive options after temporal bone resection for squamous cell carcinoma. J Plast Reconstr Aesthet Surg.

[16] Malata CM, Tehrani H, Kumiponjera D, Hardy DG, Moffat DA (2006). Use of anterolateral thigh and lateral arm fasciocutaneous free flaps in lateral skull base reconstruction. Ann Plast Surg.

[17] Teknos TN, Nussenbaum B, Bradford CR, Prince ME, El-Kashlan H, Chepeha DB (2003). Reconstruction of complex parotidectomy defects using the lateral arm free tissue transfer. Otolaryngol Head Neck Surg.

[18] Kuek LB, Chuan TL (1991). The extended lateral arm flap: a new modification. J Reconstr Microsurg.

[19] Sieg P, Hakim SG, Bierwolf S, Hermes D (2003). Subcutaneous fat layer in different donor regions used for harvesting microvascular soft tissue flaps in slender and adipose patients. Int J Oral Maxillofac Surg.

[20] Reinert S (2000). The free revascularized lateral upper arm flap in maxillofacial reconstruction following ablative tumour surgery. J Craniomaxillofac Surg.

[21] Gellrich NC, Schramm A, Hara I, Gutwald R, Duker J, Schmelzeisen R (2001). Versatility and donor site morbidity of the lateral upper arm flap in intraoral reconstruction. Otolaryngol Head Neck Surg.

[22] Depner C, Erba P, Rieger UM, Iten F, Schaefer DJ, Haug M (2012). Donor-site morbidity of the sensate extended lateral arm flap. J Reconstr Microsurg.

[23] Yu AX, Chen ZG, Yu GR. Applied anatomy of distal humerus pe-riosteo-cutaneous flap pedicled with collater-alis radialis vessels. Chin J Exp Surg. 1995;12(6):368-9.

